# 2, 3, 5, 4′-Tetrahydroxystilbene-2-O-Beta-D-Glucoside Improves Gastrointestinal Motility Disorders in STZ-Induced Diabetic Mice

**DOI:** 10.1371/journal.pone.0050291

**Published:** 2012-12-03

**Authors:** Mu-Jun Chang, Jun-Hua Xiao, Yong Wang, Yong-Li Yan, Jun Yang, Jia-Ling Wang

**Affiliations:** Department of Pharmacology, Tongji Medical College, Huazhong University of Science and Technology, Hangkong Rode, Wuhan, China; University of California, Los Angeles, United States of America

## Abstract

Oxidative stress has recently been considered as a pivotal player in the pathogenesis of diabetic gastrointestinal dysfunction. We therefore investigated the role of 2, 3, 5, 4′-tetrahydroxystilbene-2-O-beta-D-glucoside (THSG) that has a strong anti-oxidant property, in diabetic gastrointestinal dysmotility as well as the underlying protective mechanisms. THSG restored the delayed gastric emptying and the increased intestinal transit in streptozotocin (STZ)-induced diabetic mice. Loss of neuronal nitric oxide synthase (nNOS) expression and impaired nonadrenergic, noncholinergic (NANC) relaxations in diabetic mice were relieved by long-term preventive treatment with THSG. Meanwhile, THSG (10^−7^∼10^−4^ mol/L) enhanced concentration-dependently NANC relaxations of isolated colons in diabetic mice. Diabetic mice displayed a significant increase in Malondialdehyde (MDA) level and decrease in the activity of glutathione peroxidase (GSH-Px), which were ameliorated by THSG. Inhibition of caspase-3 and activation of ERK phosphorylation related MAPK pathway were involved in prevention of enhanced apoptosis in diabetes afforded by THSG. Moreover, THSG prevented the significant decrease in PPAR-γ and SIRT1 expression in diabetic ileum. Our study indicates that THSG improves diabetic gastrointestinal disorders through activation of MAPK pathway and upregulation of PPAR-γ and SIRT1.

## Introduction

Gastrointestinal (GI) motility disorders are very common in diabetic patients. Most of them suffer from associated symptoms such as reflux, early satiety, nausea, abdominal pain, diarrhea or constipation [1], [2]. The etiology of altered GI functions in diabetes is multifactorial and the mechanisms involving oxidative stress [3], [4], [5], apoptosis [6], [7], [8], neuronal loss [9], [10], [11], and advanced glycation products [12], [13], [14] are well described.

Increased oxidative stress gives rise to neuronal loss of the enteric nervous system (ENS). Damage to neurons is the main reason of GI dysmotility. Interestingly, inhibitory neurons are more affected by oxidative stress compared with excitatory neurons [15], [16], [17]. The nNOS neurons have been extensively studied in diabetic GI dysmotility. As a major NANC inhibitory neurotransmitter, nitric oxide (NO) produced by these neurons mediates the smooth muscle relaxation in the GI tract [18]. Decreased nNOS neurons and impaired NO-mediated NANC relaxation have been reported in diabetic gastroenteropathy [10], [19], [20].

More recently, oxidative stress has been recognized as an important role in GI complications of diabetes [21], [22]. Diabetes mellitus (DM) manifests a state of high oxidative stress due to hyperglycemia-induced reactive oxygen species (ROS) generation [23]. As important second messengers, ROS at low concentrations are involved in regulating apoptosis and activation of transcription factors such as nuclear factor kappa B (NF-κB). However, they can cause significant cellular damage when present in excess [24], [25]. Auto-oxidation of glucose, glucose metabolism and formation of AGEs are possible sources of ROS. Increased oxidative stress may contribute to apoptosis and the neuronal degeneration in diabetes [26], [27]. Therefore, antioxidants have therapeutic potentials for the treatment of diabetic GI motility problems [5], [21], [28].

THSG is one of the active components extracted from the traditional Chinese herb *Polygonum multiflorum*, which has been widely used as a tonic, lubricating intestine and anti-aging agent since ancient times. THSG exhibits the strong anti-oxidant and free racial-scavenging effects [29]. It has been demonstrated that THSG has a significant neuroprotective effect against ischemic brain injury in vitro and in vivo [30]. Moreover, we have previously reported an anti-inflammatory effect of THSG against experimental colitis induced by acetic acid and mitomycin C in mice [31], [32]. Thereby, all of these studies suggest that THSG may have protective effects on GI dysfunctions in diabetes. In this study, we investigated the effect of THSG on GI problems in STZ-induced diabetic mice and the underlying mechanisms.

## Results

### Effect of THSG on Fasting Blood Glucose and Body Weight in STZ-induced Diabetic Mice

Blood glucose levels in STZ-induced diabetic mice were significantly increased compared with control mice, which was not affected by THSG treatment. Body weights in diabetic mice were maintained at a significantly lower level compared with controls, which was partially ameliorated by THSG (Table 1).

**Table 1:** Effect of THSG on fasting blood glucose and body weight in STZ-induced diabetic mice.

Group	n	Blood glucose (mmol·L^−1^)		Body weight (g)	
		Start	End	Start	End
Control	24	4.9±0.3	5.0±0.2	26.0±0.5	40.3±0.7
DM	12	25.3±1.6##	27.2±1.6##	20.0±0.8##	22.1±0.8##
THSG(10 mg·kg^−1^)	11	25.4±1.5##	27.2±1.6##	19.7±0.7##	25.8±0.7##, *
THSG(30 mg·kg^−1^)	16	25.4±1.1##	27.1±1.0##	20.1±0.5##	27.4±0.7##,**
THSG(60 mg·kg^−1^)	16	25.5±1.1##	26.6±1.0##	19.8±0.5##	30.2±0.7#,**

Fasting blood glucose and body weight were measured before THSG treatment and after THSG was administrated daily for successive 8 weeks. Results are expressed as mean ± SEM.

* *P*<0.05,

** *P*<0.01, vs DM group;

# *P*<0.05,

## *P*<0.01, vs control group at corresponding time point. Control, normal control group; DM, diabetic group; THSG, THSG-treated diabetic group.

### THSG Restored the Delayed Gastric Emptying and the Increased Intestinal Transit in Diabetic Mice

**Figure 1:**
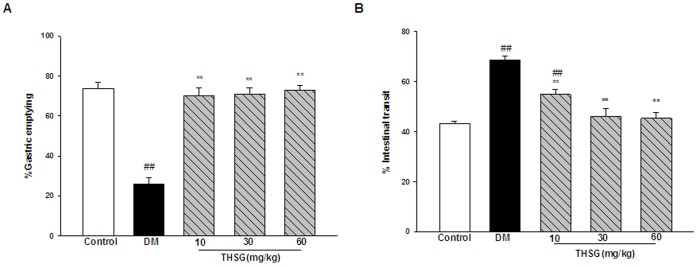
THSG restores the delayed gastric emptying and the increased intestinal transit in diabetic mice. (A) The percentage of gastric emptying. (B) Intestinal transit rate. n = 9 animals in each group. ^**^ *P*<0.01, vs DM group, ^##^ *P*<0.01, vs control group.

As shown in Figure 1A, the percentage of gastric emptying was significantly lower in STZ-induced diabetic mice compared with control animals. The rate of intestinal transit was significantly increased in diabetic group compared with controls (Fig. 1B). These alterations in gastric emptying and intestinal transit manifest the occurrence of GI dysmotility in diabetic mice. THSG restored the delayed gastric emptying and the increased intestinal transit in diabetic mice.

### THSG Improved Reduced NANC Relaxation in Diabetic Colon

**Figure 2:**
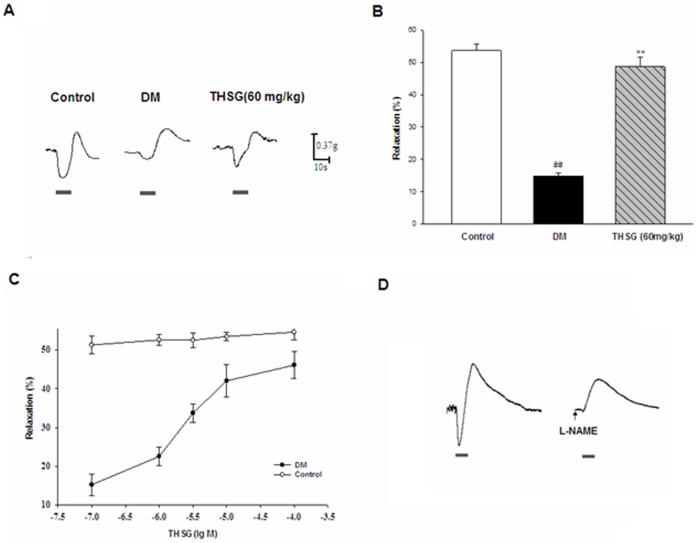
Effect of THSG on EFS-induced NANC relaxant responses in diabetic colons. (A) Representative tracing of the recording of EFS-induced relaxation. (B) NANC relaxations in colon from diabetic group were significantly reduced compared with colon from normal control group, which was partially restored by long-term (8-week) preventative THSG treatment. (C) Concentration-response curves for NANC relaxation to THSG after pre-incubation of colon strips from diabetic and normal control mouse in vitro. (D) NANC relaxations were abolished after incubation with NO synthase inhibitor L-NAME (10^−3^ M) for 1h. n = 6 animals in each group. ^**^ *P*<0.01, vs DM group, ^##^ *P*<0.01, vs control group.

In the long-term preventive study, NANC relaxations induced by electrical field stimulation (EFS) were assessed in control, diabetic and THSG-treated diabetic mice. Representative tracing of the recording of EFS-induced relaxation was shown in Figure 2A. EFS-induced NANC relaxation was significantly reduced in diabetic group compared with controls, which was partially restored by long-term (8-week) THSG treatment (Fig. 2B).

In order to further investigate the effect of THSG on colonic strips isolated from control and diabetic mice, NANC relaxations induced by EFS were recorded after pre-incubation with different concentrations of THSG (10^−7^∼10^−4^ M) in the presence of atropine, propanolol and indomethacin. As shown in Figure 2C, THSG enhanced NANC relaxation of diabetic group in a dose-dependent manner but had no obvious influence on that of controls. NANC relaxations were abolished after incubation with NO synthase inhibitor L-NAME (Fig. 2D).

### THSG Inhibited the Loss of nNOS Expression in Diabetes

**Figure 3:**
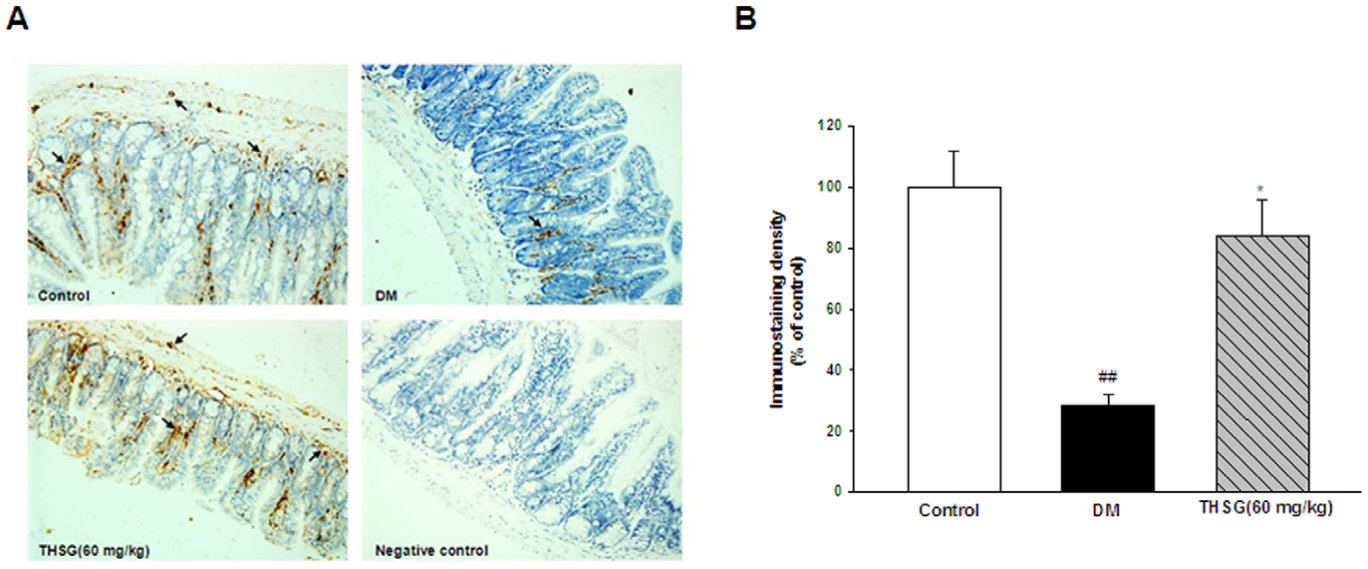
THSG prevents the loss of nNOS expression in diabetic ilea. (A) Representative photographs of sections of ilea from normal control, diabetic and THSG-treated diabetic mice stained for nNOS as well as negative control. Arrows indicate nNOS-positive area (stained brown). (B) nNOS immunostaining density was measured as mean integrated optical density. The results were expressed as percentage of control. n = 3 animals in each group. ^*^ *P*<0.05, ^**^ *P*<0.01, vs DM group; ^#^ *P*<0.05, ^##^ *P*<0.01, vs control group. Original magnification 50×.

Previous reports have demonstrated that diabetes-related GI motility dysfunction is associated with loss of nNOS neurons. Therefore, nNOS expression was assessed in ileum by using immunohistochemistry method. As shown in Figure 3A and B, nNOS expression in diabetic mice was remarkably reduced compared with control mice. THSG inhibited the loss of nNOS expression in diabetic mice.

### THSG Prevented Oxidative Damage in Diabetic Mice by Decreasing the MDA Level and Increasing GSH-Px Activity

**Figure 4:**
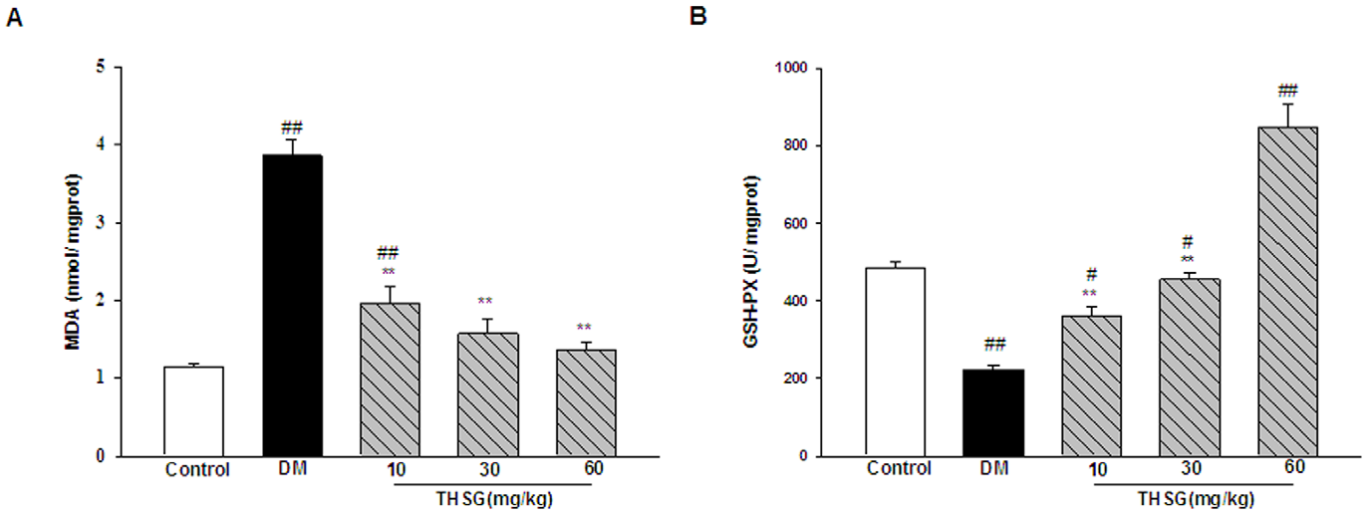
Effect of THSG on MDA level and GSH-Px activity in diabetic ilea. (A) A significant increase in MDA level was observed in diabetic mice, which was reduced by THSG. (B) THSG reversed the decrease in the activity of GSH-Px during diabetes in a dose-dependent manner. n = 6 animals in each group. ^*^ *P*<0.05, ^**^ *P*<0.01, vs DM group; ^#^ *P*<0.05, ^##^ *P*<0.01, vs control group.

A significant increase in MDA level confirms the occurrence of oxidative damage in diabetic ilea and this was dose-dependently reduced by THSG (Fig. 4A). THSG (60 mg/kg) reduced MDA level of diabetic mice to 1.36±0.11 nmol/mgprot, which was no significant difference from that of normal control (1.14±0.04 nmol/mgprot). Further, THSG reversed the decrease in the activity of GSH-Px during diabetes in a dose-dependent manner from 359.98±25.37 to 848.70±56.85 U/mgprot (Fig. 4B).

### THSG Inhibited Apoptosis in the Ilea of Diabetic Mice

**Figure 5:**
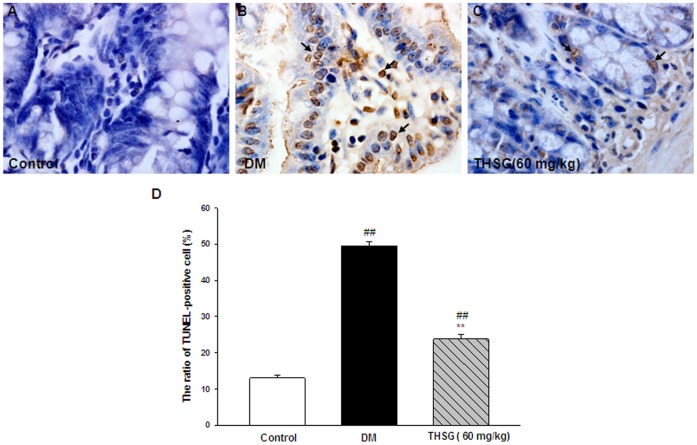
THSG inhibits apoptosis in diabetic ilea. (A, B, C) Representative photographs of TUNEL staining in the ilea. Arrows indicate TUNEL-positive cells. (D) The percentage of TUNEL-positive cells was determined as the number of TUNEL-positive cells/total number of cells in each field. n = 3 animals in each group. ^**^ *P*<0.01, vs DM group, ^##^ *P*<0.01, vs control group. Original magnification 100×.

Apoptosis can contribute to the enteric neuronal loss and disorders in GI motility in diabetes. Figure 5 showed that the number of TUNEL-positive cells was significantly increased in diabetic group (49.6±1.1%), compared with control group. Treatment with 60 mg/kg THSG could significantly reduce the number of TUNEL-positive cells in diabetic mice with the proportion of 23.8±1.4%.

### THSG Downregulated the Expression of Cleaved Caspase-3 and Upregulated the Expression of ERK1/2, PPAR-γ, SIRT1 in Diabetic Ilea

**Figure 6:**
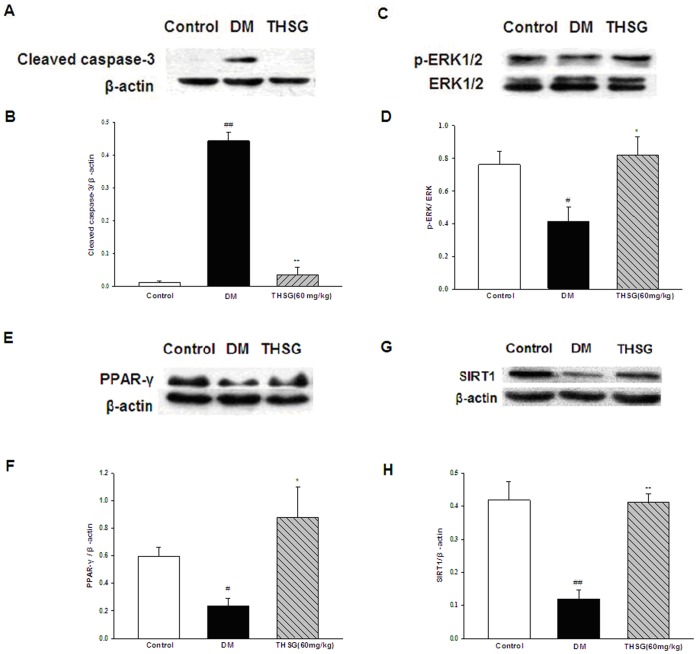
THSG downregulates the expression of cleaved caspase-3 and upregulates the expression of p-ERK1/2, PPAR-γ and SIRT1 in diabetic ilea. (A)Immunoreactive bands of cleaved caspase-3 using specific antibody. Actin was used as internal control. (B) Histogram representing the quantitative analysis of cleaved caspase-3 level normalized to actin protein. (C) Immunoreactive bands of ERK1/2 (both total and phosphorylated) using specific antibody. (D) Histogram representing the quantitative analysis of p-ERK1/2 normalized to total ERK1/2. (E) Immunoreactive bands of PPAR-γ using specific antibody. (F) Histogram representing the quantitative analysis of PPAR-γ level normalized to actin protein. (G) Immunoreactive bands of SIRT1 using specific antibody. (H) Histogram representing the quantitative analysis of SIRT1 level normalized to actin protein. n = 3 animals in each group. ^*^ *P*<0.05, ^**^ *P*<0.01, vs DM group; ^#^ *P*<0.05, ^##^ *P*<0.01, vs control group.

Caspase-3 has been identified as a key mediator in apoptosis. To examine the molecular mechanism of THSG against apoptosis in diabetic ilea, the protein levels of cleaved caspase-3, ERK1/2 (both total and phosphorylated) were measured in ileal tissues. Quality of cleaved caspase-3 protein expression was significantly increased in the diabetic ilea compared with controls, which was attenuated by THSG (Fig. 6A and B). Furthermore, p-ERK/total ERK ratio was significantly decreased in diabetic intestine, indicating decreased MAPK pathway in diabetes contributes to GI apoptosis. Antioxidant THSG could reverse a decrease in p-ERK/total ERK ratio in diabetic mice (Fig. 6C and D). In addition, diabetes also led to significant reductions in PPAR-γ and SIRT1 protein levels, and that was prevented by THSG (Fig. 6F and H).

## Discussion

Increased oxidative stress plays a key role in GI complications of diabetes, which provides us a new therapeutic strategy focused on enhancement of anti-oxidant defenses [22]. THSG is a strong anti-oxidant and free racial-scavenging agent [29]. Therefore, we examined the effect of THSG on GI problems in STZ-induced diabetic mice. The results demonstrated that THSG could restore the delayed gastric emptying and the increased intestinal transit in diabetic mice (Fig. 1), suggesting THSG has the protective effect on diabetic GI dysmotility.

Changes in enteric neuronal size and number has been observed in diabetes. Neuronal subpopulations respond differently to diabetes, which leads to the remodelling of enteric neurons. The imbalance between inhibitory and excitory neurons ratio results in impaired nerve-mediated muscle responses and GI dysmotility in diabetes. As inhibitory neurons, the nNOS neurons have been studied to a large extent in diabetes-related GI dysmotility. Several studies have demonstrated reduction in nNOS neurons and impairment in NANC relaxations both in diabetic patients and animal models [4], [10], [19]. Impairment of nitrergic function in the pylorus results in delayed gastric emptying and the loss of nitrergic neurons in the small and large intestine increases intestinal transit. There are two phases of nitrergic neuropathy in diabetes–an initial phase of loss of nNOS neurons in the content and function and a later phase with neurodegeneration [15]. The former phase can be reversed by insulin. In our study, reduced NANC relaxations and loss of nNOS expression in diabetes were relieved by THSG treatment for 8 weeks (Fig. 2B and Fig. 3), indicating that the restorative effect of THSG on diabetic GI dysmotility is probably associated with nNOS neuronal protection. Furthermore, THSG could also enhance NANC relaxations of diabetic colons in a concentration-dependent manner (Fig. 2C), suggesting that THSG may enhance NANC relaxations of diabetic mice by increasing release of NO or activating NO-related signal pathway [33].

Diabetes is associated with increased oxidative stress leading to apoptosis and neuronal degeneration. The observed significant increase in MDA level in the intestine confirms the occurrence of oxidative damage (Fig. 4A), which may bring about protein damage and inactivation of membrane bound enzymes. We also noticed a decrease in the activity of the antioxidant enzyme GSH-Px (Fig. 4B) during diabetes. THSG prevents the oxidative damage in GI tract during diabetes by decreasing the MDA level and increasing GSH-Px activity. GSH-Px activity in THSG-treated diabetic mice was significantly increased by approximately 75% over normal mice, indicating THSG may directly act on GSH-Px.

Apoptosis may be responsible for the enteric neuronal loss and abnormalities in GI motility in diabetes. Increased cleaved caspase-3 and TUNEL positive cells have been reported in the myenteric plexus of diabetic rats [6], [8]. Similar findings have been observed in our research (Fig. 6A and Fig. 5). Previous reports have demonstrated that hyperglycemia-induced reduction in the PI3K signaling in enteric neurons contributes to their apoptosis [4], [7]. As another important signal pathway for cell survival, we firstly examined the phosphorylation of ERK1/2, the most significant member of MAPK pathway in diabetic GI tract. A reduction in phosphorylation of ERK1/2 was observed in diabetic intestine (Fig. 6C and D), indicating decreased MAPK pathway in diabetes also contributes to GI apoptosis. Moreover, THSG elevated the p-ERK/total ERK ratio in diabetic intestine, suggesting that THSG inhibits apoptosis in diabetic GI tract by activation of MAPK pathway.

SIRT1 is a regulator of proteins and genes involved in the antioxidant response (FOXO3) [34], anti-apoptotic response (p53 and FOXO3) [35], [36], anti-inflammatory response (NF- κB) [37], and insulin response (IGF-1) [38]. Furthermore, SIRT1 could promote cell survival, enhance the repair of damaged DNA and reduce cell division [39], [40], [41], [42]. Activation of SIRT1 appears to be beneficial. It has been demonstrated that decreased activity of SIRT1 is associated with metabolic diseases such as obesity and type 2 diabetes [43]. Overexpression of SIRT1 and several SIRT1 activators improve glucose homeostasis and insulin sensitivity [44]. SIRT1 is considered to be a possible target for the treament of type 2 diabetes and metabolic syndrome [45], [46]. In addition, loss of SIRT1 activity has been reported in type 1 diabetic nephropathy and cardiomyopathy [47], [48]. In our study, a reduction in SIRT1 protein levels of diabetic ileum has been observed (Fig. 6G and H). THSG inhibited a decrease in SIRT1 protein levels, indicating that THSG prevents oxidative damage and apoptosis by enhancing the expression of SIRT1. Besides, we have observed that THSG-treated diabetic mice have higher survival rate than diabetic mice, which can be explained by the increased expression of SIRT1. A recent report has shown that SIRT1 plays a critical role in the control of intestinal homeostasis and gastrointestinal motility [49].

PPAR-γ participates in regulating many metabolic and inflammatory processes. Upregulation of PPAR-γ is associated with reduced oxidative stress [50], [51], [52]. In animal models of traumatic brain injury, PPAR-γ activation has been suggested to induce neuroprotection by anti-inflammatory, anti-apoptotic and anti-oxidative mechanisms [53]. Besides, PPAR-γ is a key regulator of insulin signalling, and glucose and fat metabolism. PPAR-γ agonists have been widely used in treating insulin resistance and type 2 diabetes [47]. PPAR-γ is abundant in the GI tract, especially in epithelial cells. However, the role of PPAR-γ in gut is not fully understood. In this study, we found a decrease in PPAR-γ expression in the ilea of diabetic mice (Fig. 6E and F), compared with controls, which was reversed by THSG. It suggested that upregulation of PPAR-γ expression may be one of the mechanisms by which THSG exerts its anti-oxidative and anti-apoptotic capacity.

In our study, the effect of THSG on enhancing the expression of SIRT1 and PPAR-γ may contribute to protection of diabetic GI dysfunction. Nevertheless, a previous report has shown that SIRT1 can inhibit PPAR-γ activity [54]. However, further studies will need to be performed to evaluate the interrelationship between SIRT1 and PPAR-γ.

In summary, our studies have demonstrated the beneficial effects of THSG on GI motility disorders in diabetes. And its anti-oxidative, anti-apoptotic and neuroprotective effects are probably associated with upregulation of SIRT1 and PPAR-γ. Thus, this compound may be a promising therapeutic agent for the treatment of diabetic GI dysmotility.

## Materials and Methods

### Materials

THSG was provided by the Department of Pharmacology, Tongji Medical College, Huazhong University of Science and Technology (Wuhan, China) with a purity of 99% (HPLC method). STZ was obtained from Sigma-Aldrich (St. Louis, MO, USA). The glucose kit was obtained from Biosino Biotechnology and Science (Beijing, China). Bradford and BCA protein assay kits were obtained from Beyotime Institute of Biotechnology (Shanghai, China). MDA and GSH-PX assay kits were obtained from the Nanjing Jiancheng Bioengineering Institute (Nanjing, China). The TUNEL assay kit was obtained from Roche (Mannheim, Germany). Anti-nNOS antibodies were purchased from Wuhan Boster Biological Technology (Wuhan, China). Anti-caspase-3 antibodies were purchased from Cell Signaling Technology (San Francisco, CA, USA). Anti-p-ERK, anti-ERK and anti-SIRT1 antibodies were purchased from Santa Cruz Biotechnology (CA, USA). Anti-PPAR-γ antibodies were purchased from Proteintech Group Inc. (Chicago, USA). ECL was obtained from Pierce Biotechnology (Rockford, IL, USA).

### Ethics Statement

All experimental procedures were in accordance with the Guide for Care and Use of Laboratory Animals (National Institutes of Health) and approved by the Review Committee for the Use of Human or Animal Subjects of Huazhong University of Science and Technology (Wuhan, China; permit numbers: SCXK (Hubei) 2004–0007).

### Animals

Male Kunming mice (20–25 g) were injected intraperitoneally with 150 mg/kg of STZ in 0.05 M citrate buffer, pH 4.2 or vehicle as described by Crystal C et al. [10] with some modification. This dose of STZ was selected from our preliminary experiments in which different doses of STZ (100–200 mg/kg) were tested to find the optimum dose. Five days later, blood glucose concentrations were determined in animals fasted over night by using glucose kit. Mice with fasting blood glucose levels exceeding 16.7 mmol/L were considered hyperglycemic. The rate of success in the induction of diabetes was about 88.6%. Diabetic mice were randomly divided into four groups: (1) untreated diabetic mice; (2) diabetic mice treated with THSG at three different doses (10, 30, 60 mg/kg). THSG at different doses was administrated to diabetic mice by oral gavage daily for successive 8 weeks beginning from day 7^th^ after STZ injection.

### Measurement of Intestinal Transit and Gastric Emptying

Gastric emptying and intestinal transit were determined by a method using Evans blue as described by De et al. [55]. After an overnight fast, the mice received an intragastric administration of 0.1 ml of Evans blue (50 mg in 1 ml of 0.9% sodium chloride plus 1% methylcellulose). Fifteen minutes later mice were euthanized immediately. The GI tract (stomach to cecum) was excised after careful ligation at the cardiac and pyloric ends to prevent leakage of Evans blue. The distance from the pylorus to the most distal point of migration of Evans blue was measured as well as the entire length of the small intestine. The rate of transit was calculated by the formula [(distance to Evans blue front)/(length of small intestine)] ×100.

The stomach was removed and frozen at –80°C until measurement of gastric emptying. The stomach was cut into pieces and homogenized for 30 s (Auto Science, UH-100A ultrasonic processor, 60% intensity) in 15 ml of 0.1 N NaOH. The final volume of the homogenate was adjusted to 20 ml with 0.1 N NaOH. The suspension was kept at room temperature for 1 h and then was centrifuged at 2500 rpm for 20 min at 4°C. One milliliter of supernatant was further diluted to 5 ml with 0.1 N NaOH. The absorbance of the sample was measured by UV-visible spectrophotometer (Tecan Sunrise, Tecan, Switzerland) at 570 nm (A570). The stomach and its content obtained from a mouse sacrificed immediately after oral gavage of Evans blue served as reference stomach. The percentage of gastric emptying was determined as [(A570 reference − A570 sample)/A570 reference] × 100.

### Organ Bath Physiology

Longitudinal muscle strips were isolated from the proximal colons of mice. Organ bath contained Krebs’ solution at 37°C and continuously bubbled with 95% O_2_, 5% CO_2_. Strips were mounted between two parallel platinum electrodes with one end tied to a hook at the bottom of electrode holder and the other to a force transducer. After equilibrated in Krebs’ solution for 1 h with 0.5 g of tension applied, the colonic strips were pre-incubated with atropine (1.0 µM), propanolol (1.0 µM), and indomethacin (10.0 µM) for 30 minutes to block cholinergic-, adrenergic-, and prostaglandin-mediated responses, respectively. NANC relaxations were then induced by EFS (40 V, 10 Hz, 5 ms pulse for duration of 10 s). NANC relaxations were recorded in control, diabetic and THSG-treated diabetic group (60 mg/kg, ig, for 8 weeks). In some experiments, colonic strips from control and diabetic group were pretreated with different concentrations of THSG (0.1, 1, 3, 10, 100 µM) before NANC relaxations were evoked by EFS. NANC relaxation rate was calculated as described previously [7]. Strips were incubated with NO synthase inhibitor L-NAME (10^−3^ M) before EFS to confirm NO dependence of NANC relaxations.

### Immunohistochemistry

Ilea adjacent to the cecum were fixed in 4% paraformaldehyde, embedded in paraffin wax and cut into 5 µm-thick sections on microtome. Thereafter, the sections were dried in an oven at 60°C for 30 min. The sections were heated in microwave for 10 min to rehabilitate antigens after de-paraffinized in xylene and dehydrated with graded alcohol. Subsequently, the sections were treated for 10 min with 3% hydrogen peroxide to block endogenous peroxidase activity and 30 min at 37°C with goat serum in order to reduce nonspecific staining. The sections were then incubated with the primary antibody (nNOS, 1∶100 dilution) overnight at 4°C. Before incubated with the second antibody for 30 min at room temperature, tissue sections were washed with phosphate-buffered saline (PBS). Next, the sections were washed with PBS and incubated with StreptAvidin–Biotin Complex (SABC) for 30 min at 37°C. Finally, the labeling was visualized with 3, 3′-diaminobenzidine (DAB) and hydrogen peroxide. Six selected fields per mouse were evaluated from 3 animals in each group. The nNOS-positive area was identified by a brown stain. Immunostaining density was measured as mean integrated optical density. The results were expressed as percentage of control.

### Markers of Oxidative Stress

Ilea were flushed with ice-cold 0.9% sodium chloride solution. A 10% (W/V) homogenate was prepared in saline, and then centrifuged at 3500 rpm for 15 min at 4°C. The supernatant was utilized for MDA and GSH-PX assays. The protein content was determined according to the method of Bradford using bovine serum albumin as the standard. MDA content was expressed as nmol of MDA/mg protein and GSH-PX activity was expressed as U/mg protein.

### Tunel Labeling Apoptosis

TUNEL assay was performed according to the manufacturer’s instructions. The paraffin-embedded ilea tissues were sectioned and mounted on glass slides. Briefly, after de-paraffinized and dehydrated, the slides were treated with protease K for 20 min at room temperature. Slides were then incubated with TUNEL reaction buffer in a humidified chamber at 37°C for 1 h, followed by washing with PBS. Next, slides were treated with converter-POD for 30 min at room temperature. To visualize TUNEL-positive cells, slides were stained with DAB. Sections were later washed, counterstained with haematoxylin and dehydrated before mounting. Six selected fields per mouse were evaluated from 3 animals in each group. The number of total cells and TUNEL-positive cells and the percentage of TUNEL-positive cells were calculated.

### Western Blot Analysis

Ilea were rinsed with chilled 0.9% sodium chloride solution, and homogenized in chilled lysis buffer containing 1 mM PMSF. The protein concentrations were determined by BCA protein assay kit. 8% and 12% SDS-PAGE gel were used to separate different range of proteins. Samples (40-µg protein) were separated by electrophoresis and then transferred to PVDF membranes. The membranes were blocked by 5% nonfat milk in tris-buffered saline containing 0.1% Tween-20 (TBST) for 1 h at room temperature. The membranes were incubated with appropriate primary antibodies (anti-cleaved caspase-3, anti-p-ERK, anti-ERK, anti-PPAR-γ, anti-SIRT1 and anti-β-actin antibodies) for overnight at 4°C with gentle shaking, followed by four 10-minute washes with TBST. The membranes were incubated with secondary antibody at a dilution of 1∶10,000 in TBST for 1 h at room temperature and washed four times with TBST. All visualization of immunoreactive proteins was carried out using an ECL kit.

### Statistical Analysis

Results are expressed as mean ± SEM. Data were analyzed using SPSS Version 11.5 software. Statistical differences were estimated with ANOVA followed by Student’s t-test, and *P<*0.05 was considered to be statistically significant.
